# Editorial: Precision medicine in sarcomas: the road to an effective biomarker-driven-care strategy

**DOI:** 10.3389/fonc.2023.1194593

**Published:** 2023-04-05

**Authors:** Samia Arifi, Anastasia Constantinidou

**Affiliations:** ^1^ Medical Oncology Department, Hassan II University Hospital, Fez, Morocco; ^2^ Faculty of Medicine, Pharmacy, and Dental Medicine of Fez, University of Sidi Mohamed Ben Abdellah, Fez, Morocco; ^3^ Medical School, University of Cyprus, Nicosia, Cyprus; ^4^ Medical Oncology, Bank of Cyprus Oncology Center, Nicosia, Cyprus; ^5^ Cyprus Cancer Research Institute, Nicosia, Cyprus

**Keywords:** precision medicine, sarcoma, biomarker, immunotherapy, radiomic

Sarcomas represent a rare and diverse group of mesenchymal tumors, comprising over 100 entities. This heterogeneity is further compounded by a variety of clinical presentations, including age of onset, anatomical site, and genetic predisposition. Advancements in molecular pathology have been essential in improving the clinical management of sarcomas. These advancements have allowed more accurate diagnosis and classification, as well as the identification of new potential targets and biomarkers that have enabled the development of more tailored treatment strategies, and more refined clinical trials.

Multidisciplinary management of sarcoma patients within expert centers has been critical in achieving significant clinical improvement in patients’ outcomes. Collaborative efforts between institutions and researchers have played an important role in conducting randomized clinical trials in this rare disease, and in generating large national and international databases.

Today, the management of localized sarcomas is significantly improved, primarily due to the refinement of surgical techniques that aim to balance organ preservation with R0 resection. However, it should be noted that despite achieving initial local control in many cases, distant recurrence is inevitable and associated with a poor overall prognosis. Consequently, a better understanding of individual immune, genomic, and epigenomic factors and their interactions could lead to improved outcomes and a reduced likelihood of drug resistance.

This special issue aimed to explore the unique challenges to a biomarker-driven care strategy in sarcoma patients. The collection of articles in this topic research captures the need for a synergistic development of biological and radiological biomarkers in sarcomas.

Biopsy-based tumor characterization is currently considered the standard of care for sarcoma diagnosis. However, this approach has limitations such as tumor heterogeneity, procedure-related risks, and the somewhat variable reproducibility of many pathological parameters, including grade, histologic response, round cell percentage, and surgical margins. “Virtual biopsies” have the potential to objectively and non-invasively probe tumor biology both spatially and temporally, making them a promising tool for informing clinical decisions. In their article, Arthur et al., provide a comprehensive review on imaging biomarkers (IB) studies involving sarcoma patients, stress their limits and discuss further efforts and solutions. The authors draw a hypothetical care pathway that incorporates “virtual biopsy” at two stages in the management of sarcoma patients. At diagnosis, to predict malignancy, distinguish different histotypes, and enhance grading accuracy. During treatment and monitoring, “virtual biopsy” may improve patient risk stratification, predict therapeutic response, and provide non-invasive measures of response. However, before reaching clinical practice, efforts are required in order to improve the quality of sarcomas radiomic studies and determine IBs that meet intra- and multi-centric repeatability, reproducibility, specificity, consistency, and temporality.

Thrussell et al., conducted a prospective, single site, radiomic study using MRI in patients diagnosed with retroperitoneal soft tissue sarcoma (STS). This study demonstrates good baseline repeatability of radiomic analysis based on apparent diffusion coefficient (ADC). However only a subset of radiomic features exhibited significant changes after treatment. Future multicenter studies are required in order to investigate the reproducibility of these radiomic features across different imaging centers, and validate radiomic features that showed sensitivity to post treatment changes as predictive biomarkers to response in retroperitoneal STS.

The unfulfilled need for identifying predictive biomarkers for personalized treatment in sarcomas is also well illustrated through two review articles that accompany this editorial. The first paper, by Fleuren et al., provides a critical assessment and a systematic comparison of the clinical outcomes of different anti-angiogenic multi-receptor tyrosine kinase inhibitors (RTK) in osteosarcoma (OS) and ewing sarcoma (ES). In this review, the authors assessed data on efficacy and toxicity of six drugs; pazopanib, sorafenib, regorafenib, anlotinib, Lenvatinib and cabozantinib. Altogether, these drugs, alone or in combination with other drugs, showed some efficacy across unselected patients’ populations. However, the progression free survival was improved only modestly. Currently, it is uncertain which drug would be most effective for each patient or subtype since the molecular inhibition profiles of these multi-RTK inhibitors largely overlap. Additionally, treatment resistance occurs almost uniformly, making it challenging to determine the optimal treatment approach.

The second review article by Pilavaki et al., presents the state of the art of immunotherapy in sarcoma patients. Different approaches that target the immune system have been covered in this article, including immunomodulating antibodies, adoptive cellular therapy, cancer vaccines, and cytokines. The effectiveness of immunotherapy in sarcoma seems to be depending on histology. Alveolar soft tissue part sarcoma for example, may have a meaningful response to immune checkpoint inhibitors. Conversely, conflicting results have been reported for leiomyosarcoma and undifferentiated pleomorphic sarcoma (UPS). In synovial sarcomas, the role of immune checkpoint inhibitors is limited but interestingly, the role of adoptive cellular therapy is promising. For osteosarcoma, the use of mifamurtide, an innate immunity modulator, is approved in Europe for patients with non-metastatic OS, and the combination of anti-PDL-1 and TILs therapy has yielded promising results in metastatic OS. The reasons why different sarcoma subtypes, may respond differently to immunotherapy remain unclear. Several predictive biomarkers have been investigated including microsatellite instability (MSI), mismatch repair deficiency (dMMR), tumour mutation burden (TMB), PD-L1 expression, infiltration of TILs, B cell-related gene signature, and presence of intratumoral tertiary lymphoid structures (TLSs). However, none of these biomarkers have translated into meaningful clinical players.

In conclusion, the concept of personalized therapy has become a critical component of modern cancer care, including sarcoma, which is a highly heterogenous group at all levels, from molecular subgroups to clinical presentations. Ongoing research is likely to uncover more data that will help understand the obstacles on the road to an effective biomarker-driven-care strategy for sarcoma patients.

## Author contributions

SA wrote the first draft of the editorial. AC contributed to the review and the editing. All authors contributed to the article and approved the submitted version.

